# Case report: post-salmonellosis abscess positive for *Salmonella* Oranienburg

**DOI:** 10.1186/s12879-022-07217-5

**Published:** 2022-04-05

**Authors:** Brenda M. Castlemain, Brian D. Castlemain

**Affiliations:** 1 University of New Mexico, 2211 Lomas Blvd NE, Albuquerque, NM 87106 USA; 2 New Mexico Heart Institute, University of New Mexico, 2211 Lomas Blvd NE, Albuquerque, NM 87106 USA

**Keywords:** *Salmonella* Oranienburg, Extraintestinal, Abscess, Bacteremia, Turtle

## Abstract

**Background:**

*Salmonella* gastroenteritis is a self-limited infection in immunocompetent adults. *Salmonella* Oranienburg is a serovar that has recently caused outbreaks of gastroenteritis traced to contact with a pet turtle. Extraintestinal focal infections (EFIs) with invasive *Salmonella* have been reported uncommonly, examples of which include mycotic aneurysm and spinal osteomyelitis.

**Case presentation:**

The patient is an otherwise healthy 39-year-old male with sleep apnea presenting with pain and swelling in the left anterior chest wall several months after an episode of *Salmonella* gastroenteritis and bacteremia which was treated successfully with intravenous (IV) antibiotics. He was found to have a costochondral joint abscess with operative cultures positive for *S.* Oranienburg*,* a serovar reported to have been associated with pet turtles and onions in recent CDC and FDA news releases. Of note, the joint abscess began development 2–3 months after his episode of *Salmonella* bacteremia. At the time of surgical treatment, nearly 6 months had passed since the initial episode of gastroenteritis and bacteremia.

**Conclusions:**

Delayed development of a sternocostal joint abscess after *Salmonella* bacteremia in an otherwise healthy adult male is an unusual presentation. The patient had two different exposures: a fast food chicken lunch and a pet turtle at home. Extraintestinal focal infections with invasive *Salmonella* are very uncommonly reported in healthy adult patients treated in developed countries. To our knowledge, we report this sequala for the first time.

## Background

Non-typhoidal *Salmonella* (NTS) infection generally results in a self-limited diarrheal disease in immunocompetent patients. Invasive disease (iNTS) is uncommon in developed countries but is highly reported in parts of sub-Saharan Africa. It is estimated that about 3.4 million cases of iNTS occur annually with around 681,316 deaths, 2/3 of which are children under the age of 5 [1]. *Salmonella* bacteremia is rare in immunocompetent patients, with an estimated 5% of patients developing this complication [2, 3]. Extraintestinal *Salmonella* infection is even more uncommon, though several cases have been reported- the majority in children or immunocompromised adults [4–8]. The development of a retroperitoneal abscess in a pediatric patient has been reported and speculated to have been the result of *Salmonella* penetration of the intestinal wall and lymphatic travel to the retroperitoneal nodes [5]. Here we describe a *Salmonella* infected sternocostal joint abscess that began development 2–3 months after *Salmonella* gastroenteritis treated successfully with intravenous antibiotics in an otherwise healthy patient.

## Case presentation

The patient is a 39-year-old male with medical history of obstructive sleep apnea who presented in February of 2021 with progressive left anterior chest wall pain and a fluctuant mass in that region. The patient, who had a pet turtle at home, developed crampy abdominal pain and diarrhea about 12 h after eating a fast-food chicken meal in September 2020. Within a week of that episode, he was admitted at a local hospital with fever and chills and admitted to be treated for *Salmonella* Oranienburg sepsis. Sensitivities were found to be to ampicillin, ceftriaxone, ciprofloxacin, levofloxacin and trimethoprim/sulfamethoxazole. He was treated with a 5-day course of IV levofloxacin and metronidazole, then a 10-day course of twice-daily ciprofloxacin. He recovered, but in November of 2020, he noticed progressive discomfort in the left anterior chest wall with point tenderness in the region of the fourth sternocostal joint. Anti-inflammatory medications reportedly kept the pain manageable, but he noted a gradually increasing firm but fluctuant lump in that area.

**Fig. 1:**
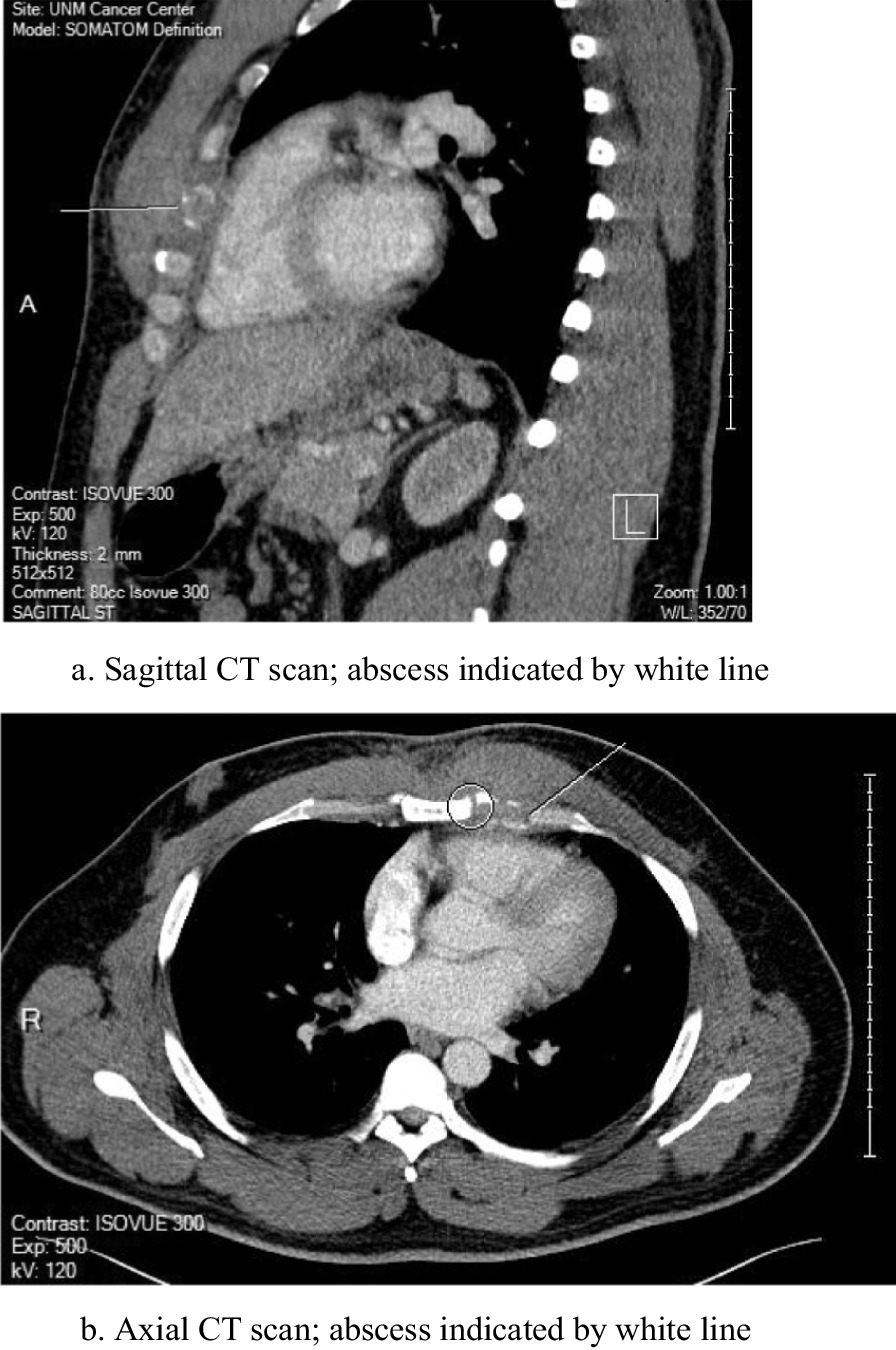
**a** Sagittal CT scan; abscess indicated by white line. **b** Axial CT scan; abscess indicated by white line

A CT scan of the chest was performed (Fig. 1a and b) and the patient was referred to the cardiothoracic surgery service for evaluation. Needle aspirate of the area failed to reveal a bacterial source or other histological abnormality, and the patient was taken to the operating room for exploration. At operation, a small discrete purulent collection was noted deep to the fourth sternocostal joint and wide debridement was undertaken with wound vac placement. Operative cultures revealed *S. Oranienburg*. Sensitivities for this isolate included ampicillin, ceftriaxone and sulfamethoxazole/trimethoprim. These isolates were not genetically compared to the blood sample taken from the bacteremia episode. The patient received IV ceftriaxone through a peripheral line for 6 weeks following his surgery. The wound closed within a month, and the patient made a full recovery several months after his surgery. Of note, the patient did not have positive stool cultures at any point during his illness.

## Discussion

Despite advances in individual and community-based sanitation, salmonellosis remains an endemic problem in most industrialized countries. The CDC estimates *Salmonella* cases at 1.35 million a year in the United States. Among those cases, there are 26,500 hospitalizations and 420 deaths [9]. Food is the source of infection in the majority of those cases. Eggs and poultry seem to be the more common food vectors, but most contaminated food items can become transmission agents.

The CDC has investigated multiple *Salmonella* outbreaks in the United States in which contact with a turtle was a common exposure [9]. In fact, small pet turtles are reported by the CDC as the source of a *Salmonella* outbreak every year for the past 5 years. [10] The 2019 pet turtle outbreak identified *Salmonella* Oranienburg as the culprit serovar [10, 11]. A recent outbreak concluding in October of 2021 was traced to onions from a single farm in Mexico and *Salmonella* Oranienburg was identified as the source of outbreak [12, 13]. Other sources of recent outbreak include salami sticks, Italian-styled meats, seafood and pre-packaged salads.

*Salmonella* infection most commonly presents as a gastroenteritis, but subacute and remote sequelae have a vast array of presentations. Immunocompromised individuals may be at increased risk for serious initial infection and long-term sequelae. Also at risk are patients in developing countries where sanitation measures and healthcare access are limited, posing barriers to effective antibiotic treatment. Extraintestinal focal infections (EFI) of NTS have been reported uncommonly with unclear risk factors. Several examples of EFIs include mycotic aneurysm, pleuropulmonary infection and spinal osteomyelitis [2]. A *Salmonella* Enteritidis infected sternal abscess was reported in a healthy 6-year-old, but this patient had no gastrointestinal symptoms before or during their disease course [14].

The case reported here is notable for several reasons. To begin, the patient is a young and healthy individual with no known history of gastrointestinal disease. His invasive infection with *Salmonella* was treated with a full course of antibiotics and the patient had negative blood cultures upon discharge. His chest wall discomfort did not begin until 2–3 months after completion of his antibiotic therapy. By the time he underwent surgical debridement, nearly 6 months had passed since his initial presentation with *Salmonella* bacteremia. We speculate that a small focus of bacteria seeded the costochondral joint space during initial infection and grew slowly in the low-oxygen environment. However, the time course is befuddling, and to our knowledge, a novel presentation. Although the pet turtle exposure is likely coincidental, we acknowledge here the relationship between *Salmonella* Oranienburg and small shelled reptiles. The pet turtle was not tested for *Salmonella* and could be a source of reinfection.

The patient in our study consumed a fast-food chicken meal 1 week prior to the onset of his gastrointestinal symptoms. Although this exposure was most likely the vector, other sources of infection must be considered*. Salmonella* was found to be present in 80% of water samples taken from the rivers of Culiacan Valley in Northwestern Mexico, with *Salmonella* Oranienburg being the most frequently isolated serotype [15]. The patient in our study resides in Alamogordo, New Mexico, home to the Tularosa basin of the Rio Grande river. While *E* Coli has been identified as the most prominent bacterium in Rio Grande waters, *Salmonella* contamination forces, such as agriculture and human waste, can be at play in any large body of water [16]. It is certainly a possibility that the patient in our case encountered a waterborne source of contamination. Other contaminated food items could have caused the infection. Although less likely, physical contact with the pet turtle without proper hand washing could also have introduced the bacterium.

In searching the literature, we encountered very few cases describing extraintestinal focal infections with *Salmonella* occurring in a developed hospital setting in a young, healthy adult individual without comorbid health conditions. A case out of Australia described a young, healthy individual with hemorrhagic cystitis caused by *S.* Oranienburg, with exposure likely resulting from *Salmonella* gastroenteritis from an extended trip to South America. Of note, the spread from the gastrointestinal to genitourinary system, particularly in the case of profuse diarrhea, is not uncommon [17–19]. Similarly, another case reports the development of an *S*. Oranienburg positive renal abscess an immunocompetent child in Taiwan. Again, this child was reported to have gastrointestinal symptoms 1 week prior to increasingly severe flank pain and fever, indicating that *Salmonella* gastroenteritis resulting in ascending genitourinary tract was likely the source of infection [20]. Our case describes a route of spread that cannot be traced with such ease. In addition, the prolonged period between initial infection with *Salmonella* and the appearance of a costochondral mass suggests that the two episodes could possibly be unrelated.

This study is limited by our need to speculate—we are unsure of the pathogenesis of the abscess described and can only formulate a hypothesis. The study is also limited by the extended period of time between the initial presentation with gastroenteritis and the described presentation with chest wall discomfort. We are not privy to any extraneous risk factors the patient may have encountered. For example, the patient may have been scratched by his pet turtle unknowingly, thus inoculating the skin with bacteria. The patient reported minimal contact with the turtle, but we cannot be certain. With recent news releases detailing outbreaks of *Salmonella* Oranienburg, we believe our case report is timely and relevant.

Our patient is a healthy 39-year-old male without significant medical illness or ongoing conditions that would have contributed to his infection. His family did have a pet turtle with which he had minimal contact. The inciting event which exposed him to the bacteria seems to have been eating chicken at a fast-food restaurant from which he developed gastroenteritis-like symptoms shortly thereafter. Of note, an interesting sequela of our patient’s infection was his development of a sternocostal joint abscess 2 or 3 months after his initial bout of infection. To our knowledge, we report this particular sequela here for the very first time. He was apparently asymptomatic from the time of his initial infection to the time period where he developed the somewhat fluctuant mass up his chest wall. The infection was treated with open debridement and IV antibiotics. The patient made a full recovery and is infection-free and doing well over a year from his initial infection.

## Data Availability

Data sharing is not applicable to this article as no datasets were generated or analyzed during the current study.

## Abbreviations

EFI: Extraintestinal focal infections
IV: Intravenous
CDC: Center for Disease Control and Prevention
FDA: Food and drug administration
NTS: Non-typhoidal *Salmonella*
iNTS: Invasive non-typhoidal *Salmonella*
CT: Computed tomography
